# Complex evolution and epidemiology of Dobrava-Belgrade hantavirus: definition of genotypes and their characteristics

**DOI:** 10.1007/s00705-012-1514-5

**Published:** 2012-10-23

**Authors:** Boris Klempa, Tatjana Avsic-Zupanc, Jan Clement, Tamara K. Dzagurova, Heikki Henttonen, Paul Heyman, Ferenc Jakab, Detlev H. Kruger, Piet Maes, Anna Papa, Evgeniy A. Tkachenko, Rainer G. Ulrich, Olli Vapalahti, Antti Vaheri

**Affiliations:** 1 Institute of Virology, Helmut-Ruska-Haus, Charité Medical School, Berlin, Germany; 2 Institute of Virology, Slovak Academy of Sciences, Bratislava, Slovakia; 3 Institute of Microbiology and Immunology, Ljubljana, Slovenia; 4 National Belgian Reference Laboratory for Hantavirus Infections, Rega Institute, Katholieke Universiteit, Leuven, Belgium; 5 Institute of Poliomyelitis and Viral Encephalitides, Russian Academy of Medical Sciences, Moscow, Russia; 6 Finnish Forest Research Institute, PL 18, 01301 Vantaa, Finland; 7 Research Laboratory for Vector Borne Diseases, Queen Astrid Military Hospital, Brussels, Belgium; 8 Virological Research Group, Szentágothai János Research Center, University of Pécs, Pécs, Hungary; 9 Faculty of Sciences, Institute of Biology, University of Pécs, Pécs, Hungary; 10 Medical School, Aristotle University of Thessaloniki, Thessaloniki, Greece; 11 Institute for Novel and Emerging Infectious Diseases, Friedrich-Loeffler-Institut, Federal Research Institute for Animal Health, Greifswald-Insel Riems, Germany; 12 Department of Virology, Infection Biology Research Program, Haartman Institute, University of Helsinki, PL 21, 00014 Helsinki, Finland

## Abstract

Dobrava-Belgrade virus (DOBV) is a human pathogen that has evolved in, and is hosted by, mice of several species of the genus *Apodemus*. We propose a subdivision of the species *Dobrava-Belgrade virus* into four related genotypes – Dobrava, Kurkino, Saaremaa, and Sochi – that show characteristic differences in their phylogeny, specific host reservoirs, geographical distribution, and pathogenicity for humans.

## History of DOBV discovery and characterization

Dobrava virus was isolated more than 25 years ago from a yellow-necked mouse, *Apodemus flavicollis,* captured in Slovenia [2]. At the same time, cell-culture isolation of Belgrade virus from a patient with severe hemorrhagic fever with renal syndrome (HFRS) was reported [11]. Later, these virus isolates were found to be identical [77]. Therefore, the International Committee for Taxonomy of Viruses (ICTV) proposed the name *Dobrava-Belgrade virus* (DOBV) for this hantavirus species [10].

Soon, reports on detection of DOBV in other European countries started to appear. DOBV nucleic acid was detected by RT-PCR and sequencing in Greek and Albanian HFRS patients [1]. Using the focus-reduction neutralization test (FRNT), DOBV-neutralizing antibodies were found in patient sera from Bosnia-Herzegovina [34], in sera from patients of a retrospectively studied HFRS outbreak in Russia (1991-92 in Tula-Ryazan region [33]), in sera of two HFRS patients from Germany [38, 39], and in human sera from Estonia [35] and Slovakia [72].

Surprisingly, DOBV was then detected also in striped field mice, *A. agrarius,* trapped on the Estonian islands Saaremaa and Vormsi [55] and subsequently isolated in cell culture [41]. Other DOBV sequences, quite distinct from those of the Saaremaa isolate, were recovered from striped field mice trapped in the Kurkino region in Russia [56] and in Slovakia [72, 73]. Meanwhile, DOBV was molecularly detected in striped field mice in other European countries such as Germany [66], Denmark [44], and other regions of European Russia [27]. Corresponding DOBV genome sequences were demonstrated in HFRS patients from Germany [23] and European Russia [27].

In several countries, both yellow-necked (*Apodemus flavicollis*; Af) and striped field mouse (*A. agrarius;* Aa)-associated strains are sympatrically present, such as Slovakia [21, 73], Slovenia [5], Hungary [16, 17, 61, 65], and Croatia (62). Phylogenetic analysis has shown that the viruses from these two different hosts also form distinct evolutionary lineages [5, 21, 73]. This finding initiated a taxonomical dispute as to whether or not the Aa-associated strains represent a distinct hantavirus species called *Saaremaa virus*, SAAV [22, 26, 58, 59]. Indeed, SAAV is currently recognized as an independent virus species on the ICTV species list (http://ictvonline.org/virusTaxonomy.asp).

Here we summarize the current knowledge on phylogeny and molecular epidemiology of *Apodemus*-associated hantaviruses in Europe and propose their taxonomical classification.

## DOBV hosts and evolution

Hantaviruses are considered host-specific, usually being associated with a single species of rodents or a few closely related species as their reservoir hosts [6, 15, 18]. For example, Tula virus is associated with voles of several species, namely the common vole *Microtus arvalis*, several other *Microtus* species, and the water vole *Arvicola amphibius* [54, 60, 67, 68, 71]. Similarly, Seoul virus is associated with rats of different species, namely *Rattus rattus*, *R. norvegicus* and *R. losea* [31, 32]. Moreover, several novel hantaviruses have been detected recently in insectivores (shrews and moles) [14], and most recently, even in bats [76, 82].

Currently, mice of at least three *Apodemus* species are recognized as DOBV hosts. The yellow-necked mouse is the dominant DOBV host in South-Eastern (SE) Europe. DOBV sequences associated with yellow-necked mice have been reported from Slovenia [3, 5], Serbia and Montenegro [51, 77], Albania and Greece [1, 43, 45, 47, 48], Croatia [37, 62], and Bulgaria [53]. Intriguingly, mice of this species are present across Europe but seem to be DOBV-free in Western and Northern Europe. Besides SE Europe, DOBV-Af has been found in several countries in Central Europe such as the Czech Republic [52, 81], Slovakia [73, 83], Hungary [40, 61] and recently also in Turkey [46, 64]. In Central and Eastern Europe (Germany, Slovakia, European Russia, Hungary, Estonia, and other countries), the striped field mouse is the dominant DOBV reservoir. DOBV-positive striped field mice have also been reported in SE Europe [5, 62]. Recently, a third natural reservoir host was identified in the Black Sea region of the European part of Russia, where about 20 % of trapped Black Sea field mice of the species *A. ponticus* (a sibling species of yellow-necked mouse, J. Michaux pers. comm.) were DOBV-antigen positive and from which virus could be isolated by cell culture with lung tissue homogenate as inoculum [27, 79].

DOBV belongs to the group of Murinae-associated hantaviruses. Its close relatives are Hantaan virus (HTNV), Seoul virus, and Thailand virus from Asia. The most closely related hantavirus currently is Sangassou virus, which is found in West Africa [25, 28].

Phylogenetic analysis of the DOBV strains from yellow-necked and striped field mice occurring sympatrically in Slovenia [5] and Slovakia [21, 73] clearly showed that DOBV forms distinct evolutionary lineages according to the host species. This was clearly confirmed when virus sequences from the third host, the Black Sea field mouse, were analyzed. These lineages were called DOBV-Af, DOBV-Aa, and DOBV-Ap according to the rodent species abbreviation of their hosts [27, 30].

**Fig. 1:**
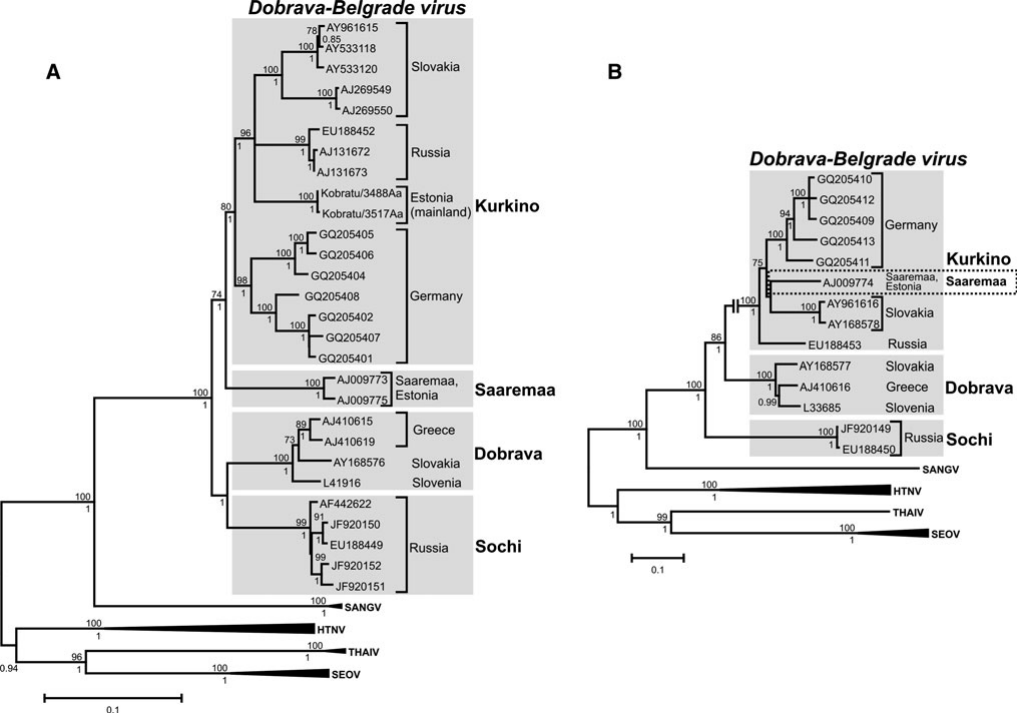
Maximum-likelihood phylogenetic trees showing the phylogenetic position of Dobrava-Belgrade virus genotypes (marked by light grey boxes) constructed on the basis of complete nucleocapsid protein (S segment) coding sequences (**A**) and complete glycoprotein precursor (M segment) coding sequences (**B**). Evolutionary analysis was conducted in MEGA5 [78]. The evolutionary history was inferred using the maximum-likelihood method based on the general time-reversible (GTR) model with a discrete Gamma distribution (+G) and five rate categories, and by assuming that a certain fraction of sites are evolutionarily invariable (+I), which was estimated to be the best-fit substitution model according to the Bayesian information criterion. The scale bars indicate an evolutionary distance of 0.1 substitutions per position in the sequence. Bootstrap values ≥70 %, calculated from 500 replicates, are shown at the tree branches, Bayesian posterior probability values ≥0.7 of the corresponding Bayesian phylogenetic tree are shown below the branches. Bayesian trees were estimated using the program BEAST with the nucleotide substitution model GTR+G+I. HTNV, Hantaan virus; SANGV, Sangassou virus; SEOV, Seoul virus; THAIV, Thailand virus

The strict host-determined differentiation is particularly obvious in the sequence analysis of the M segment, which encodes the viral envelope glycoprotein (Fig. 1B). However, in the S-segment-based trees, the virus sequences obtained from striped field mice trapped on Saaremaa Island in Estonia are clearly distinct from the other strains derived from striped field mice from mainland Europe [14, 24, 27, 53, 63]. Genetic reassortment between DOBV-Aa and DOBV-Af strains was initially proposed as a possible explanation for the conflicting S- and M-segment phylogenies [21]. Discovery of the DOBV-Ap lineage required revision of the concept. DOBV-Ap forms a sister group to DOBV-Af in the S-segment trees, and the position of the Saaremaa strain is now more ancestral [27]. Therefore, the putative reassortment could not have occurred directly with DOBV-Af as initially proposed but with some older ancestor of DOBV-Af and DOBV-Ap.

An alternative explanation based on different evolutionary rates of genome segments has been proposed [42, 59]. According to Plyusnin et al. [59], after a host switch of pre-DOBV to striped field mouse, the housekeeping nucleocapsid (N) and RNA-dependent RNA polymerase (RdRp) proteins (encoded by S and L segments) have been diverging more slowly than surface glycoproteins Gn (G1) and Gc (G2) (encoded by M segment), which are involved in the recognition of host-cell receptor(s) and represent targets for neutralizing antibodies. Consequently, the M segment has accumulated more mutations than the S (and L) segment, making phylogenetic reconstructions easier [59].

However, the proposed host switch from yellow-necked mouse to striped field mouse has been called into doubt by others [19, 26]. Indeed, high sequence variability and long branch distances among geographical clusters within the DOBV-Aa lineage indicate an isolated long-term evolution of the virus and suggest that the striped field mouse is the primary host of DOBV. On the other hand, low intra-lineage variability of DOBV-Af and DOBV-Ap strains indicates more recent and rapid spread of these viruses in yellow-necked mouse and Black Sea field mouse populations.

Additional conflicts in tree topologies suggesting genetic reassortment during DOBV evolution have been observed for DOBV-Ap. In S-segment trees, DOBV-Ap forms a well-supported sister group to DOBV-Af but is an outgroup to all other DOBV strains in M- and L-segment trees [27]. More complete sequence data, especially from M and L segments, allowing construction of more balanced datasets for all three segments, would be very helpful to better understand the complexity of DOBV evolution.

**Fig. 2:**
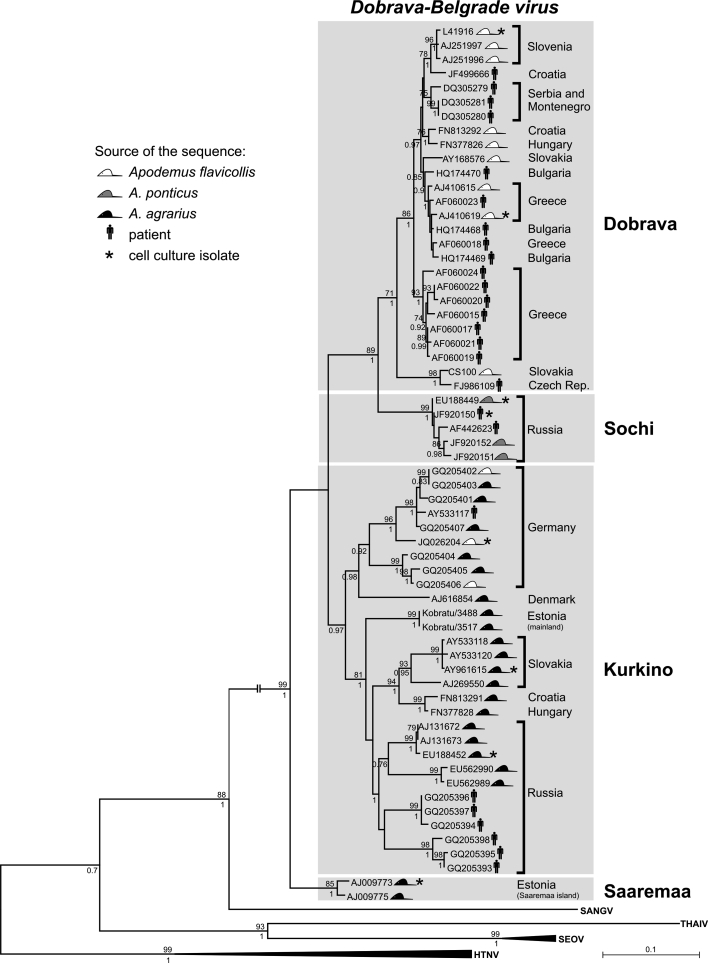
Phylogenetic tree of Dobrava-Belgrade virus strains belonging to the four genotypes (marked by light grey boxes), based on partial S segment sequences (575 bp) and showing all currently available DOBV S segment sequences of sufficient length. For methodological details of the evolutionary analysis, see legend to Fig. 1. The scale bars indicate an evolutionary distance of 0.1 substitutions per position in the sequence. Bootstrap values ≥ 70 %, calculated from 500 replicates, are shown at the tree branches; Bayesian posterior probability values ≥ 0.7 of the corresponding Bayesian phylogenetic tree are shown below the branches. Bayesian trees were estimated using the program BEAST with the nucleotide substitution model GTR + G+I. HTNV, Hantaan virus; SANGV, Sangassou virus; SEOV, Seoul virus; THAIV, Thailand virus

The inference of S-segment phylogeny seems to be particularly problematic. Usage of various datasets and phylogenetic methods can result in different positions of the Saaremaa strains [74]. Nevertheless, it remains clear that Saaremaa strains show different phylogenetic placement in S- and M-segment trees (Fig. 1) and are evolutionarily distinct from the DOBV-Aa lineage. It is important to note that the DOBV S-segment sequences obtained recently from striped field mice trapped on the Estonian mainland do not share a common ancestor with Saaremaa strains but clearly cluster with DOBV-Aa strains (Golovljova, et al., manuscript in preparation; Figs. 1A, 2). In this context, it is interesting to note that the striped field mouse population from Saaremaa Island has two pericentromeric nucleolus-organizer regions less in their karyotype than striped field mice from Estonia, Russia, or other continental areas. This has been interpreted as evidence for their earlier geographic isolation from the continental populations [7]. On the other hand, ongoing work by J. Michaux (pers. comm) shows narrow genetic diversity in striped field mice in the Western Palearctic, indicating quite recent quick expansion from the Eastern Palearctic. Due to the Ice Age, Saaremaa Island has existed for a maximum of 10,000 years, thus limiting the maximum age of the population of striped field mice found there. All of this suggests that genetic changes in these viruses can be quite fast.

Although there are difficulties in inferring phylogenetic relationships between the lineages, the following four lineages can currently be clearly recognized according to S-segment-based phylogenetic analysis: DOBV-Af, DOBV-Aa, DOBV-Ap, and SAAV (see Fig. 1A, 2). Since the appearance of SAAV in the ICTV species list, some authors designate any strain originating from *A. agrarius* as SAAV (e.g., see refs. [57, 62]) regardless of their phylogenetic distance to Estonian Saaremaa strains and to other DOBV lineages, Other authors emphasize the fact that *A. agrarius*-derived strains are not monophyletic and that DOBV-Aa strains from Central Europe, mainland Estonia and Russia are clearly different from Saaremaa Island strains. This parallel terminology has brought confusion not only to the hantavirus scientific community but also to clinicians and public-health authorities.

## Proposal of a new classification

We would like to propose a novel intra-species classification of DOBV, which is based on phylogenetic analysis of the S segment sequences. Due to the genetic basis of the classification, we propose to define virus genotypes. In agreement with the usual procedure in hantavirus terminology, genotype names should be derived from the geographical place where the first sequence of the genotype was detected.

Following this concept, one can currently define four DOBV genotypes corresponding to the four above-listed lineages. The “Dobrava” genotype consists of DOBV-Af strains and is named after the prototype virus [2]. Strains on the Estonian island of Saaremaa carried by striped field mice represent the “Saaremaa” genotype [55]. Since the first sequences of the DOBV-Aa lineage were found in the Kurkino region of Russia [56], we propose to define the “Kurkino” genotype, which corresponds to the DOBV-Aa lineage on the European mainland. Analogously, the strains of the DOBV-Ap lineage represent the “Sochi” genotype [27]. Basic characteristics of the virus genotypes are summarized in Table 1.

**Table 1:** Characteristics of virus genotypes belonging to the species *Dobrava-Belgrade virus*

Genotype	Dobrava	Kurkino	Saaremaa	Sochi
Natural host	Yellow-necked mouse *Apodemus flavicollis*	Striped field mouse *A. agrarius*	Striped field mouse *A. agrarius*	Black sea field mouse *A. ponticus*
Virus nucleotide sequences verified in patients	Yes	Yes	No	Yes
Molecular detection in rodent host in countries	Slovenia, Croatia, Greece, Czech Republic, Slovakia, Hungary, Turkey	Germany, Slovakia, Russia, Hungary, Slovenia, Croatia, Estonia (mainland)	Estonia (Saaremaa Island)	Russia
Clinical course of disease	Moderate/severe	Mild/moderate	Subclinical?	Moderate/severe
Case fatality rate	10–12 %	0.3–0.9 %	N/A	>6 %
Available cell culture isolates	Dobrava 3970/87, Belgrade Bel-1, Ano-Poroia/Af19/1999	Slovakia (SK/Aa), Aa1854/Lipetsk-02, Aa4053/Tula-02, Aa2007/Voronezh-03, EAT/Lipetsk-06, Greifswald	Saaremaa/160 V	Ap1584/Sochi-01, Sochi/hu
Prototypical virus strain and accession numbers of their genomic sequences	Dobrava 3970/87; L41916, L33685, GU904040	Slovakia/Aa; AY961615, AY961616, GU904039	Saaremaa/160 V; AJ009773, AJ009774, AJ410618	Ap1584/Sochi-01; EU188449, EU188450, EU188451
References	[1, 2, 4, 11, 40, 43, 49, 50]	[8, 16, 23, 27, 63, 66, our unpubl. data]	[13, 41]	[9, 27, 79, our unpubl. data]

N/A, data not available

Based on the recently accumulated knowledge, we are convinced that it would be more appropriate to classify these four genotypes within a single hantavirus species, *Dobrava-Belgrade virus* (DOBV). They should neither be divided into DOBV and SAAV species nor do they represent four distinct species. This opinion is based on the following four facts. (i) The amino acid sequence differences between the genotypes are extremely small, not exceeding 5 % in case of N and RdRp proteins and 10 % in case of glycoprotein precursor (GPC) (see Table 2). The current ICTV species demarcation criterion is a 7 % difference for both N and GPC amino acid sequences while a recent proposal based on similarity frequency histograms even suggests 10 % for N and 12 % for GPC sequences [36]. (ii) The genotypes cannot be distinguished using any of the routine serological methods (enzyme-linked immunosorbent assay, indirect immunofluorescence assay, immunoblot test), and in a substantial number of cases (about one-third), not even using neutralization assays, which are considered the “gold standard” for serotyping of convalescent sera [12, 24, 27, 75]. Therefore, routine serological methods can clearly identify the causative agent at the species level but not at the genotype level. Therefore, no artificial categories such as “DOBV/SAAV” need to be introduced in routine diagnostics. (iii) Spill-over infections of the DOBV-Aa strains (Kurkino genotype) to local yellow-necked mice could be observed recently in northern Germany [63, 66], and the opposite situation, the detection of the DOBV-Af strain (Dobrava genotype) in striped field mice, was observed in Croatia [62]. Such spillover infections are considered to be transient and epidemiologically irrelevant. However, they enable different hantavirus strains, or even members of different species, to “meet” in the same host, which is a basic prerequisite for genetic reassortment as well as recombination. Recently, it was shown that the genetic reassortment between the DOBV-Af lineage (defined now as Dobrava genotype) and the DOBV-Aa lineage (defined now as Kurkino genotype) can occur with high frequency in cell culture [20]. (iv) Recent discovery of the Sochi genotype in the Black Sea region shows that the list of natural hosts of DOBV still might not be complete, and novel hosts and lineages/genotypes will probably emerge in new geographical regions. If the “new host–new virus species” rule were to be followed, this could lead to claims of additional hantavirus species that could be barely differentiable from each other.

**Table 2:** Amino acid sequence differences (%) between the prototypical virus isolates of the proposed *Dobrava-Belgrade virus* species genotypes

Virus protein	Genotype*	Amino acid sequence differences (%) to genotype		
		Dobrava	Kurkino	Saaremaa
Nucleocapsid protein (S segment)	Dobrava	-		
	Kurkino	2.6	-	
	Saaremaa	3.1	3.3	-
	Sochi	2.4	2.6	3.8
Glycoprotein precursor (M segment)	Dobrava	-		
	Kurkino	6.4	-	
	Saaremaa	5.9	4.2	-
	Sochi	6.7	9.6	9.8
RNA-dependent RNA polymerase (L segment)	Dobrava	-		
	Kurkino	2.4	-	
	Saaremaa	2.7	2.7	-
	Sochi	3.4	3.6	3.6

* Sequences of the prototypical virus strains Dobrava 3970/87, Slovakia/Aa, Saaremaa/160 V, and Ap1584/Sochi-01 (for GenBank accession numbers, see Table 1), were used for calculations

## DOBV epidemiology and virulence in humans

All four DOBV genotypes have been isolated in cell culture and were molecularly detected in their respective reservoir hosts and – with the exception of the Saaremaa genotype – also in HFRS patients (Table 1). Human infections by viruses of the Dobrava genotype are mainly reported from SE Europe [4, 49], and those by members of the Sochi genotype, from the Black Sea coast region of Russia [9, 27]. However, Dobrava-genotype infections are also occasionally reported outside of SE Europe, e.g., in the Czech Republic [52], Slovakia [83, our unpublished data], and Hungary [17].

Human infections by Kurkino viruses were first reported from Germany. In accordance with the geographical distribution of *A. agrarius*, the infections are restricted to the northeastern part of the country [23, 24, 38, 39, 73]. During the period of 1991 to 2006, three large DOBV-associated HFRS outbreaks were registered in central regions of European Russia [8, 27, 33, 79]. Detailed investigation of the 2001/02 and 2005/06 HFRS outbreaks have revealed the Kurkino genotype as the causative infectious agent and the striped field mouse as a reservoir species [8, 27, 79].

So far, only three HFRS patients in Estonia have been linked by serological tests to Saaremaa (or related DOBV) infection; however, no molecular (nucleotide sequence) identification of the virus strains involved has been reported [13]. Based on the recently detected Kurkino genotype sequences in striped field mice from mainland Estonia, it seems likely that these clinical cases were caused by the Kurkino and not the Saaremaa genotype.

It is highly interesting to note that the different genotypes of DOBV– despite their high genetic similarity – induce HFRS of different severity. The most severe clinical courses were observed in SE Europe, where human infections by the Dobrava genotype occur. The case-fatality rate (CFR) of clinical cases was 10-12 %, a rate that is similar or even higher than that known for HTNV infections in Asia [4, 49, 80]. For HFRS caused by the Sochi genotype on the Black Sea coast of European Russia, a CFR of about 6 % was observed [27]; however, recent studies indicate that the CFR might be even higher (Dzagurova et al., in preparation). Whereas clinical manifestations of both Dobrava and Sochi infections are moderate to severe, the course of HFRS due to infection by Kurkino seems to be milder. During the clinically characterized large Kurkino outbreaks in European Russia in the seasons 2001/02 and 2005/06, CFRs between 0.3 % and 0.9 % were determined [8, 27]. These data confirm previous findings [38, 69, 73] that Kurkino infections cause mainly mild or moderate clinical courses of HFRS. However, during the outbreaks in Central European Russia as well as in some cases in northern Germany, severe clinical courses, even with lung impairment, were found [8, 39, 70]. In contrast, Saaremaa infections seem to be mainly subclinical. Despite the high hantaviral seroprevalence in the Saaremaa human population (28 %), no clinical cases have been reported [12, 13, 75]. At the current stage of knowledge, the order of virulence of the DOBV genotypes in humans appears to be as follows: Dobrava > Sochi > Kurkino > Saaremaa. In line with this virulence ranking in humans, a study in suckling mice demonstrated a fatal outcome for Dobrava but not Saaremaa infections [29].

It remains to be determined which genetic differences in the four virus genotypes are responsible for their different virulence. In initial investigations, we found that genetic markers associated with the divergent virulence of Kurkino (virus isolate SK/Aa) versus Dobrava (virus isolate Slo/Af), at least under *in vitro* conditions, are associated with the genomic S and L segments of the viruses [20].

## Conclusions

DOBV is the most virulent European hantavirus and is responsible for almost all fatal HFRS cases in Europe. Together with the more common, but less virulent, Puumala virus, it can be considered one of the two most important hantaviruses in Europe. Its unambiguous classification is therefore of significant benefit not only for the scientific community but also for hantavirus diagnostics, medical care, and public-health authorities. The different virulence of such closely related genotypes makes the virus particularly interesting for research. Understanding the mechanisms behind the different virulence properties of the DOBV genotypes could significantly advance the whole field of hantavirus pathogenesis.

## References

[1] Antoniadis A, Stylianakis A, Papa A, Alexiou-Daniel S, Lampropoulos A, Nichol ST, Peters CJ, Spiropoulou CF (1996). Direct genetic detection of Dobrava virus in Greek and Albanian patients with hemorrhagic fever with renal syndrome. J Infect Dis.

[2] Avsic-Zupanc T, Xiao SY, Stojanovic R, Gligic A, van der Groen G, LeDuc JW (1992). Characterization of Dobrava virus: a Hantavirus from Slovenia, Yugoslavia. J Med Virol.

[3] Avsic-Zupanc T, Toney A, Anderson K, Chu YK, Schmaljohn C (1995). Genetic and antigenic properties of Dobrava virus: a unique member of the Hantavirus genus, family Bunyaviridae. J Gen Virol.

[4] Avsic-Zupanc T, Petrovec M, Furlan P, Kaps R, Elgh F, Lundkvist A (1999). Hemorrhagic fever with renal syndrome in the Dolenjska region of Slovenia–a 10-year survey. Clin Infect Dis.

[5] Avsic-Zupanc T, Nemirov K, Petrovec M, Trilar T, Poljak M, Vaheri A, Plyusnin A (2000). Genetic analysis of wild-type Dobrava hantavirus in Slovenia: co-existence of two distinct genetic lineages within the same natural focus. J Gen Virol.

[6] Blasdell K, Henttonen H, Buchy P, Morand S, Beaudeau F, Cabaret S (2011). Hantavirus genetic diversity. New frontiers of molecular epidemiology of infectious diseases.

[7] Boeskorov G, Kartavtseva I, Zagorodniuk I, Belianin A, Liapunova EA (1995). Nucleolus organizer regions and B-chromosomes of wood mice (Mammalia, Rodentia, Apodemus) [in Russian]. Genetika.

[8] Dzagurova TK, Klempa B, Tkachenko EA, Slyusareva GP, Morozov VG, Auste B, Kruger DH (2009). Molecular diagnostics of hemorrhagic fever with renal syndrome during a Dobrava virus infection outbreak in the European part of Russia. J Clin Microbiol.

[9] Dzagurova TK, Witkowski PT, Tkachenko EA, Klempa B, Morozov VG, Auste B, Zavora DL, Iunicheva IV, Mutnih ES, Kruger DH (2012). Isolation of Sochi virus from a fatal case of hantavirus disease with fulminant clinical course. Clin Infect Dis.

[10] Fauquet CM, Mayo MA, Maniloff J, Desselberger U, Ball LA (2005). Virus taxonomy. Eighth report of the International Committee on Taxonomy of Viruses.

[11] Gligic A, Dimkovic N, Xiao SY, Buckle GJ, Jovanovic D, Velimirovic D, Stojanovic R, Obradovic M, Diglisic G, Micic J, Asher DM, LeDuc JW, Yanagihara R, Gajdusek DC (1992). Belgrade virus: a new hantavirus causing severe hemorrhagic fever with renal syndrome in Yugoslavia. J Infect Dis.

[12] Golovljova I, Sjölander KB, Lindegren G, Vene S, Vasilenko V, Plyusnin A, Lundkvist A (2002). Hantaviruses in Estonia. J Med Virol.

[13] Golovljova I, Vasilenko V, Mittzenkov V, Prükk T, Seppet E, Vene S, Settergren B, Plyusnin A, Lundkvist A (2007). Characterization of hemorrhagic fever with renal syndrome caused by hantaviruses, Estonia. Emerg Infect Dis.

[14] Henttonen H, Buchy P, Suputtamongkol Y, Jittapalapong S, Herbreteau V, Laakkonen J, Chaval Y, Galan M, Dobigny G, Charbonnel N, Michaux J, Cosson JF, Morand S, Hugot JP (2008). Recent discoveries of new hantaviruses widen their range and question their origins. Ann NY Acad Sci.

[15] Hjelle B, Yates T (2001). Modeling hantavirus maintenance and transmission in rodent communities. Curr Top Microbiol Immunol.

[16] Jakab F, Horváth G, Ferenczi E, Sebok J, Varecza Z, Szucs G (2007). Detection of Dobrava hantaviruses in Apodemus agrarius mice in the Transdanubian region of Hungary. Virus Res.

[17] Jakab F, Sebok J, Ferenczi E, Horváth G, Szucs G (2007). First detection of Dobrava hantavirus from a patient with severe haemorrhagic fever with renal syndrome by SYBR Green-based real time RT-PCR. Scand J Infect Dis.

[18] Jonsson CB, Figueiredo LT, Vapalahti O (2010). A global perspective on hantavirus ecology, epidemiology, and disease. Clin Microbiol Rev.

[19] Khaiboullina SF, Morzunov SP, St Jeor SC (2005). Hantaviruses: molecular biology, evolution and pathogenesis. Curr Mol Med.

[20] Kirsanovs S, Klempa B, Franke R, Lee MH, Schönrich G, Rang A, Kruger DH (2010). Genetic reassortment between high-virulent and low-virulent Dobrava-Belgrade virus strains. Virus Genes.

[21] Klempa B, Schmidt HA, Ulrich R, Kaluz S, Labuda M, Meisel H, Hjelle B, Kruger DH (2003). Genetic interaction between distinct Dobrava hantavirus subtypes in Apodemus agrarius and A. flavicollis in nature. J Virol.

[22] Klempa B, Ulrich R, Meisel H, Krüger DH, Schmidt HA, Kaluz S, Labuda M, Hjelle B (2003). Genetic interaction between Dobrava and Saaremaa hantaviruses: Now or millions of years ago? (Authors’ Reply). J Virol.

[23] Klempa B, Schütt M, Auste B, Labuda M, Ulrich R, Meisel H, Krüger DH (2004). First molecular identification of human Dobrava virus infection in central Europe. J Clin Microbiol.

[24] Klempa B, Stanko M, Labuda M, Ulrich R, Meisel H, Krüger DH (2005). Central European Dobrava Hantavirus isolate from a striped field mouse (Apodemus agrarius). J Clin Microbiol.

[25] Klempa B, Fichet-Calvet E, Lecompte E, Auste B, Aniskin V, Meisel H, Denys C, Koivogui L, ter Meulen J, Krüger DH (2006). Hantavirus in African wood mouse, Guinea. Emerg Infect Dis.

[26] Klempa B, Meisel H, Kruger DH, Ulrich R, Stanko M, Labuda M (2006). Saaremaa hantavirus should not be confused with its dangerous relative, Dobrava virus – Author’s reply. J Clin Microbiol.

[27] Klempa B, Tkachenko EA, Dzagurova TK, Yunicheva YV, Morozov VG, Okulova NM, Slyusareva GP, Smirnov A, Kruger DH (2008). Hemorrhagic fever with renal syndrome caused by 2 lineages of Dobrava hantavirus, Russia. Emerg Infect Dis.

[28] Klempa B, Witkowski PT, Popugaeva E, Auste B, Koivogui L, Fichet-Calvet E, Strecker T, Ter Meulen J, Krüger DH (2012). Sangassou virus, the first hantavirus isolate from Africa, displays genetic and functional properties distinct from those of other Murinae-associated hantaviruses. J Virol.

[29] Klingström J, Hardestam J, Lundkvist A (2006). Dobrava, but not Saaremaa, hantavirus is lethal and induces nitric oxide production in suckling mice. Microbes Infect.

[30] Kruger DH, Klempa B, Liu D (2011). Dobrava-Belgrade virus. Molecular detection of human viral pathogens.

[31] Lee HW, Baek LJ, Johnson KM (1982). Isolation of Hantaan virus, the etiologic agent of Korean hemorrhagic fever, from wild urban rats. J Infect Dis.

[32] Liu PQ, Liao HX, Fu JL, Hang CS, Song G (1984). Isolation of epidemic hemorrhagic fever virus from *Rattus losea* and *Rattus confucianus* and their antigenic identification. Bull Jiangxi Med Coll.

[33] Lundkvist A, Apekina N, Myasnikov Y, Vapalahti O, Vaheri A, Plyusnin A (1997). Dobrava hantavirus outbreak in Russia. Lancet.

[34] Lundkvist A, Hukic M, Horling J, Gilljam M, Nichol S, Niklasson B (1997). Puumala and Dobrava viruses cause hemorrhagic fever with renal syndrome in Bosnia-Hercegovina: evidence of highly cross-neutralizing antibody responses in early patient sera. J Med Virol.

[35] Lundkvist Å, Vasilenko V, Golovljova I, Plyusnin A, Vaheri A (1998). Human Dobrava hantavirus infections in Estonia. Lancet.

[36] Maes P, Klempa B, Clement J, Matthijnssens J, Gajdusek DC, Krüger DH, Van Ranst M (2009). A proposal for new criteria for the classification of hantaviruses, based on S and M segment protein sequences. Infect Genet Evol.

[37] Markotić A, Nichol ST, Kuzman I, Sanchez AJ, Ksiazek TG, Gagro A, Rabatić S, Zgorelec R, Avsic-Zupanc T, Beus I, Dekaris D (2002). Characteristics of Puumala and Dobrava infections in Croatia. J Med Virol.

[38] Meisel H, Lundkvist Å, Gantzer K, Bär W, Sibold C, Krüger DH (1998). First case of infection with hantavirus Dobrava in Germany. Eur J Clin Microbiol Infect Dis.

[39] Mentel R, Bordihn N, Wegner U, Wendel H, Niklasson B (1999). Hantavirus Dobrava infection with pulmonary manifestation. Med Microbiol Immunol.

[40] Németh V, Madai M, Maráczi A, Bérczi B, Horváth G, Oldal M, Kisfali P, Bányai K, Jakab F (2011). Detection of Dobrava-Belgrade hantavirus using recombinant-nucleocapsid-based enzyme-linked immunosorbent assay and SYBR Green-based real-time reverse transcriptase-polymerase chain reaction. Arch Virol.

[41] Nemirov K, Vapalahti O, Lundkvist A, Vasilenko V, Golovljova I, Plyusnina A, Niemimaa J, Laakkonen J, Henttonen H, Vaheri A, Plyusnin A (1999). Isolation and characterization of Dobrava hantavirus carried by the striped field mouse (Apodemus agrarius) in Estonia. J Gen Virol.

[42] Nemirov K, Henttonen H, Vaheri A, Plyusnin A (2002). Phylogenetic evidence for host switching in the evolution of hantaviruses carried by Apodemus mice. Virus Res.

[43] Nemirov K, Vapalahti O, Papa A, Plyusnina A, Lundkvist A, Antoniadis A, Vaheri A, Plyusnin A (2003). Genetic characterization of new Dobrava hantavirus isolate from Greece. J Med Virol.

[44] Nemirov K, Andersen HK, Leirs H, Henttonen H, Vaheri A, Lundkvist A, Plyusnin A (2004). Saaremaa hantavirus in Denmark. J Clin Virol.

[45] Olsson GE, Leirs H, Henttonen H (2010). Hantaviruses and their hosts in Europe: reservoirs here and there, but not everywhere?. Vector-Borne Zoon Dis.

[46] Oncul O, Atalay Y, Onem Y, Turhan V, Acar A, Uyar Y, Caglayik DY, Ozkan S, Gorenek L (2011). Hantavirus infection in Istanbul, Turkey. Emerg Infect Dis.

[47] Papa A, Johnson AM, Stockton PC, Bowen MD, Spiropoulou CF, Alexiou-Daniel S, Ksiazek TG, Nichol ST, Antoniadis A (1998). Retrospective serological and genetic study of the distribution of hantaviruses in Greece. J Med Virol.

[48] Papa A, Spiropoulou C, Nichol S, Antoniadis A (2000). Tracing Dobrava hantavirus infection. J Infect Dis.

[49] Papa A, Antoniadis A (2001). Hantavirus infections in Greece–an update. Eur J Epidemiol.

[50] Papa A, Nemirov K, Henttonen H, Niemimaa J, Antoniadis A, Vaheri A, Plyusnin A, Vapalahti O (2001). Isolation of Dobrava virus from Apodemus flavicollis in Greece. J Clin Microbiol.

[51] Papa A, Bojovic B, Antoniadis A (2006). Hantaviruses in Serbia and Montenegro. Emerg Infect Dis.

[52] Papa A, Zelená H, Barnetová D, Petrousová L (2010). Genetic detection of Dobrava/Belgrade virus in a Czech patient with Haemorrhagic fever with renal syndrome. Clin Microbiol Infect.

[53] Papa A, Christova I (2011). Genetic detection of Dobrava/Belgrade virus, Bulgaria. Emerg Infect Dis.

[54] Plyusnin A, Vapalahti O, Lankinen H, Lehvaslaiho H, Apekina N, Myasnikov Y, Kallio-Kokko H, Henttonen H, Lundkvist A, Brummer-Korvenkontio M, Gavrilovskaya I, Vaheri A (1994). Tula virus - a newly detected hantavirus carried by European common voles. J Virol.

[55] Plyusnin A, Vapalahti O, Vasilenko V, Henttonen H, Vaheri A (1997). Dobrava hantavirus in Estonia: does the virus exist throughout Europe?. Lancet.

[56] Plyusnin A, Nemirov K, Apekina N, Plyusnina A, Lundkvist Å, Vaheri A (1999). Dobrava hantavirus in Russia. Lancet.

[57] Plyusnin A (2002). Genetics of hantaviruses: implications to taxonomy. Arch Virol.

[58] Plyusnin A, Vaheri A, Lundkvist Å (2003). Genetic interaction between Dobrava and Saaremaa viruses: now or millions of years ago?. J Virol.

[59] Plyusnin A, Vaheri A, Lundkvist A (2006). Saaremaa hantavirus should not be confused with its dangerous relative, Dobrava virus. J Clin Microbiol.

[60] Plyusnina A, Laakkonen J, Niemimaa J, Henttonen H, Plyusnin A (2008). New genetic lineage of Tula Hantavirus in Microtus arvalis obscurus in Eastern Kazakhstan. Open Virol J.

[61] Plyusnina A, Ferenczi E, Rácz GR, Nemirov K, Lundkvist A, Vaheri A, Vapalahti O, Plyusnin A (2009). Co-circulation of three pathogenic hantaviruses: Puumala, Dobrava, and Saaremaa in Hungary. J Med Virol.

[62] Plyusnina A, Krajinović LC, Margaletić J, Niemimaa J, Nemirov K, Lundkvist Å, Markotić A, Miletić-Medved M, Avšič-Županc T, Henttonen H, Plyusnin A (2011). Genetic evidence for the presence of two distinct hantaviruses associated with Apodemus mice in Croatia and analysis of local strains. J Med Virol.

[63] Popugaeva E, Witkowski PT, Schlegel M, Ulrich RG, Auste B, Rang A, Krüger DH, Klempa B (2012). Dobrava-Belgrade hantavirus from Germany shows receptor usage and innate immunity induction consistent with the pathogenicity of the virus in humans. PLoS ONE.

[64] Sarıgüzel N, Hofmann J, Canpolat AT, Türk A, Ettinger J, Atmaca D, Akyar I, Yücel S, Arıkan E, Uyar Y, Cağlayık DY, Kocagöz AS, Kaya A, Kruger DH (2012). Dobrava hantavirus infection complicated by panhypopituitarism, Istanbul, Turkey, 2010. Emerg Infect Dis.

[65] Scharninghausen JJ, Meyer H, Pfeffer M, Davis DS, Honeycutt RL (1999). Genetic evidence of Dobrava virus in Apodemus agrarius in Hungary. Emerg Infect Dis.

[66] Schlegel M, Klempa B, Auste B, Bemmann M, Schmidt-Chanasit J, Büchner T, Groschup MH, Meier M, Buschmann A, Zoller H, Krüger DH, Ulrich RG (2009). Multiple Dobrava-Belgrade virus spillover infections, Germany. Emerg Infect Dis.

[67] Schlegel M, Kindler E, Essbauer SS, Wolf R, Thiel J, Groschup MH, Heckel G, Oehme RM, Ulrich RG (2012). Tula virus infections in the Eurasian water vole in Central Europe. Vector Borne Zoonotic Dis.

[68] Schmidt-Chanasit J, Essbauer S, Petraityte R, Yoshimatsu K, Tackmann K, Conraths FJ, Sasnauskas K, Arikawa J, Thomas A, Pfeffer M, Scharninghausen JJ, Splettstoesser W, Wenk M, Heckel G, Ulrich RG (2010). Extensive host sharing of central European Tula virus. J Virol.

[69] Schütt M, Gerke P, Meisel H, Ulrich R, Kruger DH (2001). Clinical characterization of Dobrava hantavirus infections in Germany. Clin Nephrol.

[70] Schütt M, Meisel H, Kruger DH, Ulrich R, Dalhoff K, Dodt C (2004). Life-threatening Dobrava hantavirus infection with unusually extended pulmonary involvement. Clin Nephrol.

[71] Sibold C, Sparr S, Schulz A, Labuda M, Kozuch O, Lysy J, Kruger DH, Meisel H (1995). Genetic characterization of a new hantavirus detected in Microtus arvalis from Slovakia. Virus Genes.

[72] Sibold C, Meisel H, Lundkvist Å, Schulz A, Cifire F, Ulrich R, Kozuch O, Labuda M, Kruger DH (1999). Simultaneous occurrence of Dobrava, Puumala, and Tula hantaviruses in Slovakia. Am J Trop Med Hyg.

[73] Sibold C, Ulrich R, Labuda M, Lundkvist Å, Martens H, Schütt M, Gerke P, Leitmeyer K, Meisel H, Kruger DH (2001). Dobrava hantavirus causes hemorrhagic fever with renal syndrome in central Europe and is carried by two different Apodemus mice species. J Med Virol.

[74] Sironen T, Vaheri A, Plyusnin A (2005). Phylogenetic evidence for the distinction of Saaremaa and Dobrava hantaviruses. Virol J.

[75] Sjolander KB, Golovljova I, Vasilenko V, Plyusnin A, Lundkvist Å (2002). Serological divergence of Dobrava and Saaremaa hantaviruses: evidence for two distinct serotypes. Epidemiol Infect.

[76] Sumibcay L, Kadjo B, Gu SH, Kang HJ, Lim BK, Cook JA, Song JW, Yanagihara R (2012). Divergent lineage of a novel hantavirus in the banana pipistrelle (Neoromicia nanus) in Côte d’Ivoire. Virol J.

[77] Taller AM, Xiao SY, Godec MS, Gligic A, Avsic-Zupanc T, Goldfarb LG, Yanagihara R, Asher DM (1993). Belgrade virus, a cause of hemorrhagic fever with renal syndrome in the Balkans, is closely related to Dobrava virus of field mice. J Infect Dis.

[78] Tamura K, Peterson D, Peterson N, Stecher G, Nie M, Kumar S (2011). MEGA5: Molecular evolutionary genetics analysis using maximum Likelihood, evolutionary distance, and maximum parsimony methods. Mol Biol Evol.

[79] Tkachenko EA, Okulova NM, Iunicheva IuV, Morzunov SP, Khaĭbulina SF, Riabova TE, Vasilenko LE, Bashkirtsev VN, Dzagurova TK, Gorbachkova EA, Sedova NS, Balakirev AE, Dekonenko AE, Drozdov SG (2005). The epizootological and virological characteristics of a natural hantavirus infection focus in the subtropic zone of the Krasnodarsk Territory [in Rusian]. Vopr Virusol.

[80] Vaheri A, Henttonen H, Voutilainen L, Mustonen J, Sironen T, Vapalahti O (2012). Hantavirus infections in Europe and their impact on public health. Rev Med Virol.

[81] Weidmann M, Schmidt P, Vackova M, Krivanec K, Munclinger P, Hufert FT (2005). Identification of genetic evidence for Dobrava virus spillover in rodents by nested reverse transcription (RT)-PCR and TaqMan RT-PCR. J Clin Microbiol.

[82] Weiss S, Witkowski P, Auste B, Nowak K, Weber N, Fahr J, Mombouli JV, Wolfe ND, Drexler JF, Drosten C, Klempa B, Leendertz FH, Kruger DH (2012). Hantavirus in bat, Sierra Leone. Emerg Infect Dis.

[83] Zelena H, Zvolankova V, Zuchnicka J, Liszkova K, Papa A (2011). Hantavirus infection during a stay in a mountain hut in Northern Slovakia. J Med Virol.

